# Sulfated Polysaccharides from Seaweed Strandings as Renewable Source for Potential Antivirals against *Herpes simplex* Virus 1

**DOI:** 10.3390/md20020116

**Published:** 2022-02-01

**Authors:** Hugo Pliego-Cortés, Kévin Hardouin, Gilles Bedoux, Christel Marty, Stéphane Cérantola, Yolanda Freile-Pelegrín, Daniel Robledo, Nathalie Bourgougnon

**Affiliations:** 1 Laboratoire de Biotechnologie et Chimie Marines, Université Bretagne Sud, EA3884, IUEM, 56000 Vannes, France; hugo-skyol_pliego-cortes@univ-ubs.fr (H.P.-C.); kevin.hardouin@univ-ubs.fr (K.H.); gilles.bedoux@univ-ubs.fr (G.B.); christel.marty@univ-ubs.fr (C.M.); 2 Plateforme RMN-RPE, Service Général des Plateformes, UFR Sciences et Techniques, Université Bretagne Occidentale, 6 Av. V. Le Gorgeu CS93837, CEDEX 3, 29238 Brest, France; Stephane.Cerantola@univ-brest.fr; 3 Centro de Investigación y de Estudios Avanzados (CINVESTAV), Unidad Mérida, AP 73, Cordemex, Mérida 97310, Yucatán, Mexico; yolanda.freile@cinvestav.mx (Y.F.-P.); daniel.robledo@cinvestav.mx (D.R.)

**Keywords:** *Ulva* sp., *Sargassum muticum*, *Halymenia floresii*, *Solieria chordalis*, red seaweed, Vero cells, Herpes simplex virus, polysaccharides

## Abstract

*Herpes simplex* virus 1 (HSV-1) remains a prominent health concern widespread all over the world. The increasing genital infections by HSV-1 that might facilitate acquisition and transmission of HIV-1, the cumulative evidence that HSV-1 promotes neurodegenerative disorders, and the emergence of drug resistance signify the need for new antiviral agents. In this study, the in vitro anti-herpetic activity of sulfated polysaccharides (SPs) extracted by enzyme or hot water from seaweeds collected in France and Mexico from stranding events, were evaluated. The anti-herpetic activity evaluation of the semi-refined-polysaccharides (sr-SPs) and different ion exchange purified fractions showed a wide range of antiviral activity. Among them, the sr-SPs from the Rhodophyta *Halymenia floresii* showed stronger activity EC_50_ 0.68 μg/mL with SI 1470, without cytotoxicity. Further, the antiviral activity of the sr-SPs evaluated at different treatment schemes showed a high EC_50_ of 0.38 μg/mL during the viral adsorption assays when the polysaccharide and the virus were added simultaneously, whilst the protection on Vero cell during the post-infection assay was effective up to 1 h. The chemical composition, FTIR and ^1^H NMR spectroscopic, and molecular weights of the sr-SPs from *H. floresii* were determined and discussed based on the anti-herpetic activity. The potential utilization of seaweed stranding as a source of antiviral compounds is addressed.

## 1. Introduction

*Herpes simplex* virus 1 (HSV-1) remains a prominent health concern widespread all over the world. Approximately 67% of the world’s population are living with HSV-1 infection, an equivalent estimate of 3.75 billion individuals aged under 49 years old. Infections from HSV-1 are lifelong, highly contagious, and prevalent with periodic recurrences. Asymptomatic viral shedding spreads most infections, contributing to the global prevalence [1]. Beyond the oral, pharynx, and eye infections, serious illnesses, such as blindness or hearing impairment could develop [2]. Further, observational epidemiological and biological studies have suggested that HSV-1 infection might facilitate acquisition and transmission of HIV-1 and in co-infected patients, might accelerate HIV-1 disease progression [3]. Additionally, an increasing proportion of genital infections caused by HSV-1 was estimated at 5.2% of the population between 15 to 49 years due to oral to genital contact, since they can interchangeably infect oral or genital sites [1,4]. As an alpha-herpesvirus establish latency in neurons, viral replication in the brain may produce infections of the central nervous system causing encephalitis and meningitis [5]. Currently, cumulative investigations are supporting the relationship of HSV-1 in Alzheimer’s disease (AD), and to oxidative stress conditions [6]. In humans, the production, and accumulation of anti-HSV immunoglobulins, considered as bio-markers of HSV-1 reactivation [7], and the presence of the type 4 allele of the apolipoprotein E gene APOE-ε4 [8], have been correlated with an increased risk of AD. HSV has developed sophisticated and highly efficacious strategies to escape immune recognition and to subvert host response [3]. Medications available for systemic treatment of HSV are acyclovir (ACV) and its analogs, such as famciclovir, valacyclovir, and penciclovir, act by interfering with viral DNA polymerase, preventing viral replication [9]. However, the increase in the drugs resistance, toxicity, and adverse effects of these treatments, coupled with the actual incidence of virus emergence and epidemics, stimulates the need for new, safe, and efficient antiviral agents to reduce the impact of viral infections on human populations [10,11].

Derived compound from natural products has historically been the basis of many drug discoveries. Seaweeds have proven to be exceptionally rich sources for natural active molecules, such as polysaccharides. The structurally and chemically complex cell wall of the seaweeds contains different classes of polysaccharides highly diverse in composition, molecular masses, or the presence of functional groups, among others, and depending on the species, life-cycle stage, geographic location, or season, could represent up to 60% of the dry weight (dw). These polysaccharides are made up of monomers linked by glycosidic bonds [12]. In a broad context, the cell walls consist of a fibrillary neutral skeleton (cellulose) and an amorphous embedding matrix. This matrix in red seaweeds (Rhodophyta) are rich in sulfated galactans, as agars and carrageenans, and in brown seaweed (Ochrophyta) presents carboxylic alginate and fucans and fucoidans’ fucose-containing sulfated polysaccharides (FCSP), and in green seaweeds (Chlorophyta) the most studied polysaccharides are the ulvans [13]. Some of them are industrially exploited and used as food additives like thickeners and emulsifiers.

Over the past few decades, experimental trials have evidenced the potential antiviral that seaweed sulfated polysaccharides (SPs) exert against a wide spectrum of viral infections for human pathogens such as human immunodeficiency virus [14,15,16], papillomavirus [17], or poliovirus [18], among others. In vitro research on HSV-1 has shown important anti-herpetic activity from SPs; in our research group, some species of seaweeds and classes of SPs have been tested [19,20,21,22,23,24,25], and similarly, activity from unusual SPs from the Rhodophyta *Schizymenia* species [18,26], hybrids and complex carrageenans [27,28,29], fucoidans [23,30,31], and ulvans [25,32,33]. The efficacy of these SPs mainly corresponds to the ability to block the virus attachment to the cell surface. According to the viral infection cycle, very few SPs have shown virucidal activity [30,34], and inhibition during the replication [35,36,37]. In addition, despite having good inhibitory effects on virus infection, the high molecular weight (MW) and poor tissue-penetrating ability of polysaccharides have limited potential antiviral application in humans. Oligosaccharide prepared from polysaccharides by chemical or enzymatic degradation has smaller molecular weight and is easy to contact with viruses, thus bioavailability and biological activity increased [38,39].

It is highly necessary to explore all the possibilities to obtain antiviral agents from natural sources in order to develop novel types of antiviral drugs, which allow a combined therapy to treat HSV infections by targeting different steps in the virus life-cycle, such as the entry inhibitors that have recently emerged as new promising drugs [40]. Thus, scientific knowledge in the search for natural products with the potential to produce possible antiviral agents (i.e., oligosaccharides), able to inhibit viral infections is substantial; it is even known that natural products present a broad antiviral spectrum and have the advantage to reduce drug resistance and secondary undesirable effects, since current approved drugs are completely synthetic. Likewise, the discovery of new antivirals should represent low production costs and must come from renewable natural sources, such as seaweeds. This source can be easily obtained from the huge under-utilizable biomass from blooms and stranding events, which constitutes economic and ecologic constraints. Additionally, seaweed biomass can be produced on a large-scale farming system [41]. 

Within these frames of reference, the aim of this research was to evaluate the antiviral activity in vitro of SPs at different treatment schemes against HSV-1, extracted by the eco-friendly process of enzyme-assisted extraction from seaweeds exclusively collected from mass blooms and stranding events on the Mexican Caribbean and the French Brittany coasts.

## 2. Results

### 2.1. Enzyme-Assisted Extraction Increased the SPs Yields

The yield of the semi-refined sulfated polysaccharides (sr-SPs) isolated by ethanol and dialysis was significantly higher (*p* < 0.05) in all the enzyme-assisted extractions (EAE) compared to the hot water extraction (HWE), except for the brown seaweed *Sargassum muticum*. The highest yields were found in the Chlorophyta *Ulva* sp. and the Rhodophyta *Solieria chordalis* with 29.3 ± 0.1 and 19.8 ± 0.7% of dry weight (dw), respectively (Figure S1). The purification by ion exchange resin yielded three fractions (F1, F2, and F3). The specific yield for F1 and F3 was lower than 5% in all samples, while F2 showed specific yields in a range of 14 to 19.8% dw. No significant differences (*p* > 0.05) were found among EAE and HWE (data not shown).

### 2.2. Screening for Antiviral Activity and Cytotoxicity of Sulfated Polysaccharides

**Figure 1:**
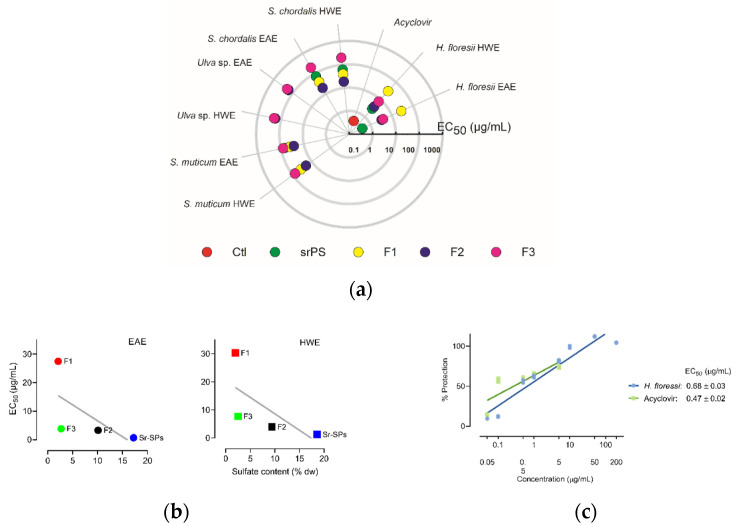
Antiviral activity (EC_50_ μg/mL) of sulfated polysaccharides against HSV-1 after 72 h infection at MOI 0.001 ID_50_/cells. (**a**) Screening of anti-herpetic compounds. Polar plot direction goes from high anti-herpetic activity (lower EC_50_ values) in the center to low anti-herpetic activity (higher EC_50_) at the end. Ctl: positive control corresponds to acyclovir; sr-SPS: semi-refined sulfated polysaccharides obtained from enzyme-assisted extraction (EAE) or hot water extraction (HWE) and their purified fractions, F1, F2, F3; (**b**) antiviral activity (EC50 μg/mL) in the function of sulfate content of *H. floresii* for the different fractions from EAE and HWE, the gray line represent the linear regression (*r*^2^ = 0.40 and 0.46 respectively); (**c**) percentage of protection on Vero cell from *H. floresii* semi-refined polysaccharide in the function of concentration. Data are the mean of duplicates (*n* = 2), full data available in Supplementary Table S1.

The in vitro screening allowed to identify the anti-herpetic activity among the different SPs, semi-refined and purified fractions (F1, F2, F3). As shown in Figure 1a, most of the extracts exhibited antiviral activity against *Herpes simplex* virus 1 (HSV-1), except for *Ulva* sp. with very low activity, even in the higher concentration of 200 μg/mL. Likewise, all F3 fractions and *H. floresii* F1 were less active. A significant (*p* < 0.05) increase in the antiviral activity was observed in the EAE compared to HWE in the sr-SPs for *H. floresii* and *S. chordalis*. The purification process for *S. muticum* F2 and *S. chordalis* F2 increased by 3-fold the activity compared to sr-SPs extracted by both enzyme and hot water. The red seaweed *Halymenia floresii* exhibited the higher antiviral activity (EC_50_ μg/mL) among the different species studied. The EC_50_ in the function of sulfate content of *H. floresii* for the different fractions from EAE and HWE is shown in Figure 1b, and the sr-SPs and F2 showed the higher content of sulfate groups with the higher antiviral activity in both EAE and HWE (*r*^2^ = 0.40 and 0.46). An effective percentage of protection on the infected Vero cells, given a potent anti-herpetic activity in the EAE (EC_50_ 0.68 μg/mL), compared to acyclovir (0.47 μg/mL) since no statistically differences were observed (*p* < 0.05) between them (Figure 1c).

The cytotoxicity determined by cell viability with the neutral red dye assay on Vero cells showed a low percentage of destruction (<10%) at the higher concentration of 1000 μg/mL (Table S2). Further, neither negative effects, such as microscopically alteration of normal cell morphology nor the destruction of the cell layer was observed.

### 2.3. Antiviral Activity at Different Treatment Schemes from Selected Polysaccharides

The antiviral activity of *H. floresii* for both EAE and HWE sr-SPs, and Fractions 2 of purified-SPs from *S. muticum* and *S. chordalis* showed higher antiviral activity during the screening study. Therefore, the antiviral activity of these SPs was studied at different treatment schemes: pre-treatment of the cells with SPs, pre-treatment of the virus with SPs, viral adsorption assays, and post-infection assays.

**Figure 2:**
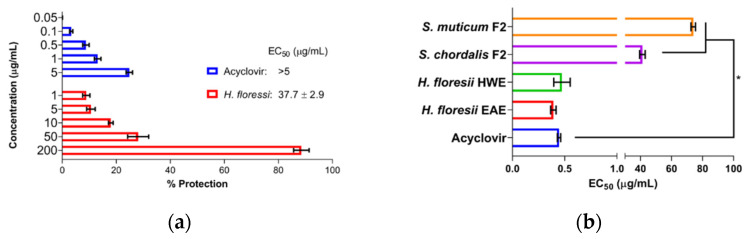
Antiviral activity under different treatment schemes at MOI 0.001 ID_50_/cells, (**a**) the pre-treatment of the cells with semi-refined sulfated polysaccharide (sr-SPs), sr-SPs from *H. floresii* obtained by enzyme-assisted extraction (EAE); (**b**) the pre-treatment of the virus with sr-SPs. HWE: hot water extraction. F2: Fraction 2 was obtained from the ion exchange purification of EAE. Data are the mean ± SD (*n* = 2). * Significance difference (*p* < 0.05) according to Kruskal–Wallis (pair-wise comparison).

The pre-treatment of the cells with SPs showed that sr-SPs from *H. floresii* extracted by both EAE and HWE conferred low protection of Vero cells against HSV-1 infection (EC_50_ 37.7 ± 2.9 and 48.0 ± 0.3 μg/mL), and were higher than *S. chordalis* and *S. muticum* SPs. The control (acyclovir) showed a reduced percentage of protection according to the concentrations tested (Figure 2a).

The pre-treatment of the virus with SPs showed high activity when Sr-SPs from *H. floresii* were pre-incubated directly with the virus suspension with an effective concentration EC_50_ of 0.39 ± 0.02 and 0.47 ± 0.07 μg/mL. Additionally, acyclovir showed an EC_50_ of 0.45 ± 0.01 μg/mL. The purified-SPs from *S. muticum* and *S. chordalis* showed a significantly lower activity (*p* < 0.05) compared to acyclovir (Figure 2b).

The viral adsorption assays showed that SPs were effective when added simultaneously with the virus suspension (TA) and when they were added at the same time and after the infection (TC). The efficacy of *H. floresii* sr-SPs obtained by EAE were higher in TA (EC_50_ 0.38 ± 0.06 μg/mL) and comparable to acyclovir (0.42 ± 0.04 μg/mL) since no significance difference (*p* > 0.05) were observed. However, when sr-SPs were added after viral adsorption, the protection was reduced up to 1.76 ± 0.2 μg/mL for *H. floresii*, while the inhibition of the virus adsorption for the p-SPs from *S. chordalis* and *S. muticum* were almost null (Table 1, virus adsorption).

In the post-infection assay, higher antiviral activity was observed for *H. floresii* EAE samples, when added simultaneously and 1 h after viral infection (EC_50_ 1.2 ± 0.4 and 2.4 ± 0.1 μg/mL). All antiviral activity against viral infection decreases considerably over time. The purified-SPs from *Sargassum muticum* showed very low inhibition until 3 h post-infection and for *S. chordalis* the activity was not detected from 2 h post-infection. On the contrary, the drug reference remained significantly (*p* < 0.05) active up to 5 h post-infection (Table 1, post-infection).

### 2.4. Biochemical Composition of Purified Polysaccharides

The biochemical composition of the purified sulfated polysaccharides (p-SPs) Fraction 2 is shown in Table 2. The neutral sugar content was higher in *H. floresii* EAE (37% dw) and *Ulva* sp. HWE (28.4% dw). Polysaccharides from *Solieria chordalis* and *Sargassum muticum* showed the higher amount of sulfate groups (15.4% and 12.9% dw), while *Ulva* sp. polysaccharide showed up to 17% dw of uronic acids. The 3,6-anhydrogalactose (3,6-AG) in *S. chordalis* was 8.8% dw and only 0.5% dw in *H. floresii*. Protein content was variable among species and ranged from 1.1% dw in *H. floresii* to 7.5% dw in *S. chordalis*. Fractions 1 and 3 showed a low content of sulfate groups with less than 3% dw, while the neutral sugars ranged from 35 to 45% dw (Table S3).

**Figure 3:**
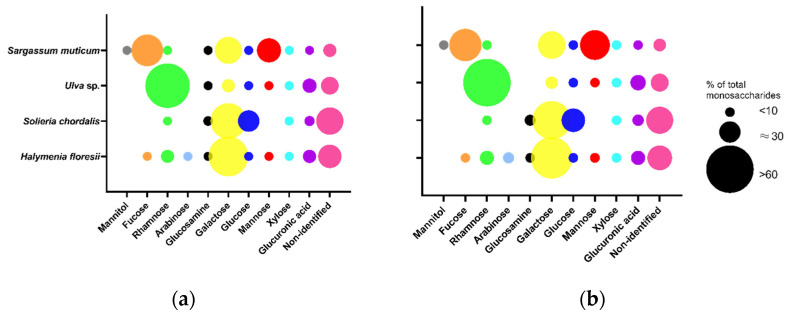
Monosaccharide composition of purified sulfated polysaccharides (p-SPs) Fraction 2 was obtained by (**a**) enzyme-assisted extraction (EAE) or by (**b**) hot water extraction (HWE). Data are the mean of duplicates (*n* = 2). Circle sizes represent the percentage of each monosaccharide according to the legend. Full data in Supplementary Table S4.

The total content of monosaccharides determined in Fraction 2 ranged from 107 to 142 μg/mg of dw in the different species. The red seaweeds *H. floresii* (Figure 3) and *S. chordalis* were rich in galactose (57.8 and 46.5% of total content), whilst in *Ulva* sp. and *S. muticum* the main monosaccharide was rhamnose and fucose, respectively (Table S4). The profile of monosaccharides from *Sargassum muticum* allowed identifying these polysaccharides as fucose-containing sulfated polysaccharides (FCSP).

### 2.5. Preliminary Characterization and Molecular Mass of H. floresii Polysaccharides

Based on the higher antiviral activity, the semi-refined sulfated polysaccharides from *Halymenia floresii* were studied for their biochemical composition and chemical structure by FT-IR and NMR spectroscopy, and their molecular weight was evaluated. The biochemical composition (Table 3) of the semi-refined sulfated polysaccharide from both EAE and HWE were similar, and no significant differences (*p* > 0.05) were observed. 

**Figure 4:**
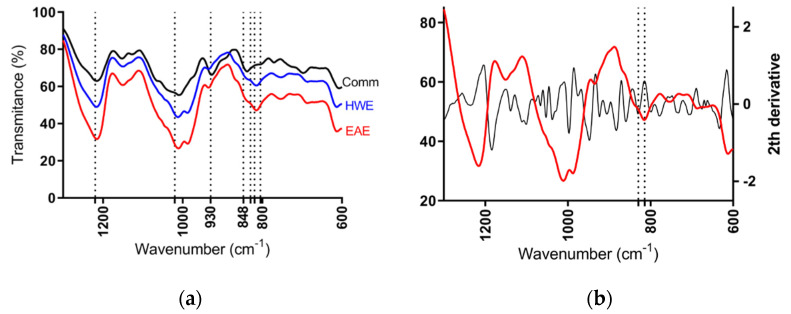
FT-IR spectrum of semi-refined sulfated polysaccharides of (**a**) *H. floresii* EAE (enzyme-assisted extraction), HWE (hot water extraction), and Comm (commercial sample); (**b**) Second derivative of sr-SPs of *H. floresii* EAE.

According to the FT-IR spectra of semi-refined sulfated polysaccharide from *H. floresii*, samples were determined as λ-carrageenan type when compared to the commercial λ-carrageenan spectra, and the information available in the published literature on the same species [42]. No differences on spectra were observed between EAE and HWE, not even between the purified and semi-refined samples (Data not shown). All spectra showed the band of sulfate esters at 1210–1250 cm^−1^, and a broad band around 1020 cm^−1^ corresponding to non-gelling carrageenan types. The band at 928–933 cm^−1^ attributable to 3,6-anhydrogalactose residues was weak in both EAE and HWE extracts, but evidently in the commercial sample. The characteristics bands of ι- and κ-carrageenans at 848 cm^−1^ assigned to d-galactose-4-sulphate (G4S), and at 805 cm^−1^ assigned to d-galactose-2-sulphate (DA2S) typical of ι-carrageenan were completely absent in the *H. floresii* extracts. However, the commercial carrageenan showed a strong band at 840 cm^−1^ (Figure 4a). It is important to highlight, that carrageenan from *H. floresii* showed a broad band between 830–820 cm^−1^. Which, in the second derivative spectra, this broad band showed two well-defined bands at 829.8, assigned for G2S, and at 814.5 cm^−1^ corresponding to DA2S (Figure 4b), which may suggest the occurrence of a hybrid carrageenan.

**Figure 5:**
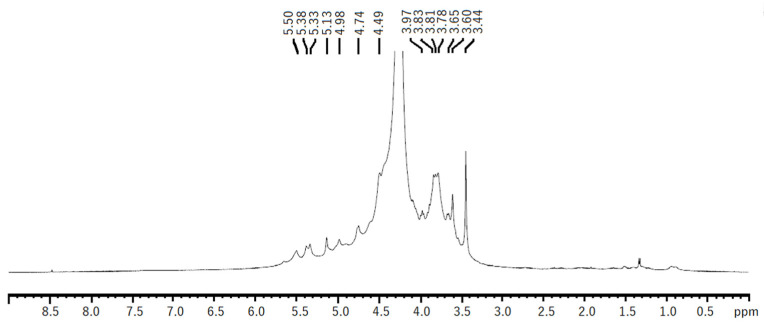
^1^H NMR spectrum of native polysaccharide from *H. floresii* obtained by hot water extraction.

The ^1^H NMR spectra of both EAE and HWE were similar. The spectra showed some chemical shifts, which could be related to the λ-carrageenan polysaccharide previously reported in *Iridaea undulosa* [43] and *Gigartina skottsbergii* [44]. The signals for the α-anomeric protons referred to (1,4)-linked galactopyranoses (D-unit) were observed at 5.50–5.51 ppm corresponding to D26S H1. While signals related to (1,3)-linked β-d-galactopyranoses (G-unit) were observed at 4.74 and 4.49 ppm, which could be related to G2S protons H1, H2, respectively (Figure 5). Other non-identified chemical shifts were observed, however, they could be related to other types of carrageenans, such as hybrid carrageenans. 

During additional solubilization assays, the commercial lambda-carrageenan and the semi-refined and purified sulfated polysaccharides, both EAE and HWE from *H. floresii*, were soluble in 0.3 M KCl solution and were recovered by precipitation with isopropanol. On the contrary, commercial kappa and iota carrageenan were insoluble in 0.3 M KCl and were not recovered after alcohol precipitation.

**Figure 6:**
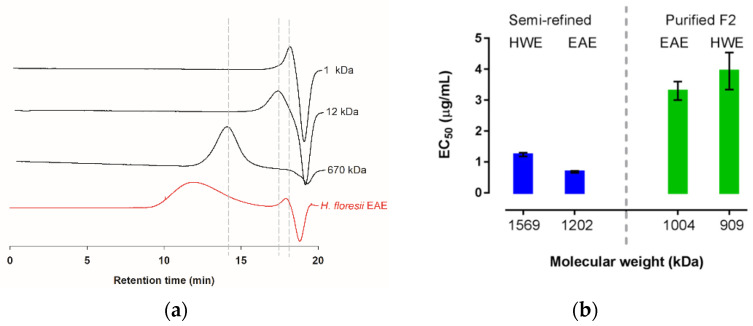
Molecular weight distribution of λ-carrageenan from *H. floresii* (**a**) Chromatograms extracted from HPSEC of the semi-refined samples obtained by enzyme-assisted extraction (EAE) and dextran sulfate as standards (1, 12 and 670 kDa); (**b**) Antiviral activity (EC_50_ μg/mL) as a function of estimated molecular weight for semi-refined and fraction 2 (F2) purified by anion exchange.

The weight average molecular masses (AMw) of the semi-refined polysaccharide obtained from the EAE and the HWE were determined through the intrinsic viscosities yielding 1202.8 and 1569.8 kDa, respectively (Table 4); the commercial sampled of lambda-carrageenan showed an AMw of 688.6 kDa. Further, based on the high-performance steric exclusion chromatography (HPSEC), this commercial λ-carrageenan was calculated as 732.9 kDa. However, the carrageenan of *H. floresii*, both EAE and HWE, showed a broad peak ranging from minute 10 to 16, and only in the EAE chromatogram, a small peak of about 4.4 kDa was observed (Figure 6a), according to the distribution of the dextran sulfates standards. The broad peaks were out of range of the linear equation and therefore were not calculated by HPSEC.

The molecular weight of the carrageenans from *H. floresii*, including the semi-refined extracted by both EAE and HWE, and their respective ion exchange purified Fraction 2, are presented in Figure 6b. In terms of EC_50_, the antiviral activity was higher for the MW of 1202 kDa and 1569 kDa, corresponding to the semi-refined carrageenans extracted by the enzyme and hot water. 

## 3. Discussion

### 3.1. The Enzyme-Assisted Extraction Produced Higher Yields of Polysaccharides

The enzyme-assisted extraction (EAE) is a green environmentally friendly extraction method known for its high efficiency and reduced solvent consumption and time. EAE has proven to efficiently increase the yields of specific compounds, which usually is done by solvents or strong alkali. Enzymes are allowed to recover the compounds and maintain their bioactivity. Commercial enzymes (i.e., Alcalase 0.8 L FG, Protamex, Glucanase, Celluclast 1.5 L FG) are commonly used, since specific enzymes for seaweeds, such as carrageenase [39,45], fucanase, or alginate-lyase [46], is limited and they are not commercially available yet [47]. However, using the specific enzymes for degradation of polysaccharides could be a key step in the production of bioactive oligosaccharides. Recently, the λ-carrageenase from *Pseudoalteromonas carrageenovora* was isolated and characterized [41]. Authors reported the production of the oligosaccharides neo-λ-carratetraose and neo-λ-carrahexaose. Thus, this enzymatic process could avoid the disadvantages of chemical modification, such as the decrease or loss of specific groups (i.e., sulfate groups) and therefore, the loss of bioactivity. In this study, the increased yields of sulfated polysaccharides of *Halymenia floresii*, *Solieria chordalis*, and *Ulva* sp. were observed; but not for *Sargassum muticum*. Our results are in agreement with a previous study, reporting that, from the brown seaweed *Fucus evanescens*, a lower fucoidan yield was obtained by EAE compared to classical extraction [46]. This could be explained by the use of acidic solution (HCl) which leads to better cell wall hydrolysis [47]. On the other hand, the higher yields observed in the red and green seaweeds validated the efficiency of EAE, such as for *Ulva armoricana* by using a neutral and alkaline endo-proteases extraction yield up to 88.4% in the amount of solubilized dry matter [33], while the isolation of ulvans from *Ulva* sp. by ethanol precipitation and dialysis yielded 24.6% of the initial dry matter using the Protamex enzyme [48], being similar to this study for *Ulva* sp. (29.3% dw). Therefore, the recovery of target compounds by EAE should be done based on the characteristics of species, the enzymes, and the parameters of extraction, since all these aspects will influence the biochemical composition. Burlot et al. [22], revealed that optimized extraction conditions (i.e., hydrolysis time or enzyme concentration) had significant positive effects on sulfated polysaccharides from *S. chordalis*. We observed the higher sulfate groups (12–15% dw) and protein content (6–7.5% dw) were extracted in *S. muticum* and *S. chordalis*. Since composition was lower than 100%, it was possible the presence of mineral matter (not determined) which were not totally removed through the dialysis process, such as [48] reported for *Ulva* sp., could result in a 7% reduction of mineral matter after ethanolic precipitation and up to 60% after 7 days of dialysis. In general, no significant differences were registered on monosaccharides composition, except for glucuronic acid in *Sargassum muticum*. In addition, a higher recovery of fucose was achieved by EAE being comparable to the classical mild-acid extracted (HWE), and a very low amount of mannitol was observed; this is explained because the FCSPs are complex polysaccharides with high diversity on their structures, and could present glucuronic acid, xylose, mannose, and rhamnose [49]. While, the low content of glucose could be due to a co-precipitated of residues of cellulose due to the incomplete degradation during the extraction process. In this context, the efficacy on extraction must impact the potential utilization of seaweed blooms and stranding as antiviral compounds.

### 3.2. Antiviral Activity of Sulfated Polysaccharides

The screening of the anti-herpetic test revealed a wide range of activity due to the diversity of the polysaccharide composition and structures, which included ulvans, fucans, and galactans. Among them, the purified SPs from *Solieria chordalis* and *Halymenia floresii* were found to significantly inhibit the in vitro infection of *Herpes simplex* virus 1 (HSV-1), with EC_50_ values of 18.4 and 3.3 with a CC_50_ of 200 μg/mL, therefore the selective indexes of 10.8 and 60.6, respectively. The antiviral activity has been related to the content of sulfate groups, the sulfation patterns, and the molecular weight or chemical composition. The sulfate content exerts the antiviral activity because of the capacity to interfere with the initial attachment of the herpes virus to the cells [2]. In this sense, the lower antiviral activity of the SPs from *Ulva* sp. could be related to the low content of sulfate groups (ca. 5%), as previously reported on [33] for *Ulva armoricana*, and due to the low anti-herpetic activity, we did not include in the study of the SPs at different treatment. On the other hand, a previous report on ι-carrageenan rich in sulfate groups (25% dw) and 3,6-AG (30% dw) from *S. chordalis* obtained by microwave-assisted extraction showed 10-folds higher anti-herpetic activity (EC_50_ 1.8 μg/mL) than classical HWE tested under the similar condition as the present study [24]. In this study, the sulfate content in function of the antiviral activity for the different fractions showed the higher EC_50_ for the fractions with higher content of sulfate groups, however, the lower *r*^2^ (0.40 and 0.46) could be explained since beyond a certain limit the amount of sulfate groups (around 5%) is no longer the only parameters limiting the antiviral activity of the polysaccharides. The carrageenan of *H. floresii* (F2) showed higher anti-herpetic activity and lower sulfate groups compared to polysaccharides from *S. chordalis* (9% vs. 15% dw), this activity is also related to the distribution of sulfate groups in the backbone, and may allow the forming of a stable polysaccharide-virus complex [50]. A strong anti-herpetic activity (IC_50_ 0.3 to 0.7 μg/mL) was reported for the red algae *Gigartina skottsbergii*, on their λ-carrageenan fractions 1T, 1T1, 1T2 and the hybrid µ/ν-carrageenan 1C3. This was attributed to the sulfate groups localized in a similar position to those of the sulfate groups of the α-D-galactose 2,6-disulfate units from heparan sulfate [27,34], a well study molecule that interacts with glycoproteins of the HSV [2]. At the same time, the position of sulfate group at C2 in β-galactose units of their cyclized derivatives [27]. Native λ-carrageenan of *H. floresii* is a d-galactose with a sulfate group at C2 (β-unit) linked to a d-galactose sulfated at C2 and C6 in α-unit [42]. Furthermore, ι-carrageenan from *S. chordalis* is a galactose with a sulfate group at C4 linked to one anhydrogalactose sulfated at C2 [51]. According to the 3,6-AG obtained in our results, *S. chordalis* contained 8.4% dw and H. *floresii* 0.6–1.1% dw, Carlucci et al. [27], suggested that the decreases in the chain flexibility increases the antiviral activity, since chain flexibility increases with the percentage of 3,6-AG our results are in agreement, being *H. floresii* more active. 

The monosaccharides may also influence the antiviral activity, as reported for ι-carrageenan with a low molecular weight rich in galactose with high antiviral activity in *S. filiformis* [52]. In our study, the carrageenans from *H. floresii* and *S. chordalis*, and the FCSP from *S. muticum* showed a high content of galactose (57.8%, 46.5%, and 29.6% of total monosaccharides, respectively). Likewise, enhanced antiviral activity was related to the higher content of glucose (55.9%) from an extract of *Codium fragile* [21]. A previous study has shown that the production of infectious HSV-1 was reduced up to 98% by growth in 6 mM 2-deoxy-d-glucose [53]. Our results showed the highest content of glucose in *S. chordalis*. As a general tendency, the EAE exhibited an increasing antiviral activity in all seaweed tested except on the purified fraction of *Sargassum muticum*. Protease enzymes have been reported to efficiently break the cell walls and promote access to the internal content of the carrageenophyte *Solieria* species [22,52], *Chondrus crispus*, *Codium fragile* [21], and *Ulva* [25,33], in which the anti-herpes activity was increased in the enzyme extracts comparing to the HWE. The low antiviral activity in *S. muticum* could be related to the lower content of sulfate groups compared to other species, for instance, fractions Shap-1 and Shap-2 from *S. henslowianum* showed activity against HSV-1 with EC_50_ 0.89 and 0.82 μg/mL, attributed mainly to the high content of sulfate groups 31.9% dw and fucose 76.3% [47].

Despite the purification process, this has been shown to enhance the antiviral activity against HSV in Fraction 2 for *S. muticum* and *S. chordalis*, as well as on other species of seaweeds [26,36]. A potent anti-herpetic activity was observed in the semi-refined carrageenan of *H. floresii* obtained by EAE, with 17% dw of sulfate groups, that showed an EC_50_ value of 0.68 μg/mL and a selective index of 1470 since no cytotoxicity was observed up to 1000 μg/mL (Supplementary Table S2). This anti-herpetic carrageenan was comparable to the drug reference, acyclovir with EC_50_ 0.43 μg/mL and a SI of 2325. Some reviews have addressed the strong and broad-spectrum antiviral of the different types of carrageenans [11,32,50,54,55]. Nevertheless, to our knowledge, no studies have been published on this anti-herpetic polysaccharide extracted by an enzymatic eco-friendly process from *Halymenia floresii* obtained from under-utilizable stranding events, suggesting a promising biomaterial with antivirals against HSV-1, which could be used to further produce antiviral agents, such as oligosaccharides. 

According to the antiviral activity tested at different treatment schemes, during the pre-treatment of the cells with SPs, no activity for carrageenan from *S. chordalis* and FCSP from *S. muticum* was observed and very low activity for carrageenan from *H. floresii*. However, the antiviral activity of this carrageenan observed during the pre-treatment of the virus with SPs was higher. This activity is related to the inhibition of the virus/cell interactions and could be similar to other sulfated polysaccharides, such as heparin, an SPs with an extracellular mechanism that shows an inhibitory effect for a variety of viruses by interacting with their surface, and the broad antiviral activity of heparin that has allowed the development of numerous heparin-mimetic compounds [56]. Carrageenans from *G. atropurpurea* [30] and *G. skottsbergii* [34] showed higher antiviral activity than heparin. Further, the protection of the cytopathic effect produced by HSV-1 on HeLa cells was 5-fold higher in commercial ι-carrageenan than heparin [35].

During the viral adsorption assays, TA and TC showed the higher inhibition of the viral adsorption for *H. floresii* EAE when the SPs was added at the same time of infection, the EC_50_ was lower than acyclovir (0.38 vs. 0.42 μg/mL), without significance difference. Similarly, when polysaccharide or acyclovir was added before and after the adsorption period, a stronger activity was detected (EC_50_ 0.18 vs. 0.09 μg/mL). However, in TB, when SPs was added after the adsorption period, the antiviral activity was reduced in both EAE and HWE (EC_50_ of 1.7 and 7.6 μg/mL). Our results are in agreement with those studies where antiviral activity was mainly ascribed to the inhibition of adsorption during the initial steps of infection, including complex carrageenan-agar [26] and hybrid carrageenans [27,28,29].

The post-infection assays revealed a moderated antiviral activity of the carrageenan from *H. floresii* EAE. The Vero cells were protected only in the first hour after infection. On the other hand, *S. chordalis* and *S. muticum* SPs presented a very low activity. The carrageenan extracted from *Stenogramme interrupta* [28], *G. skottsbergii* [34], and *Gigartina acicularis* [57] interfered efficiently during the early steps of the viral replication. In our study, the antiviral activity at this step could be related to the presence of the small peak with a molecular weight estimated of 4.4 kDa, since low molecular weight compounds (LMWC) have shown activity at the intracellular level. The LMWC 3-*O*-sulfated octasaccharide obtained from heparin had strong activity in blocking HSV-1 infection [58], and the magnesium-modified heparin show a highly non-specific transduction inhibition on HSV-1 [59]. Further studies are required to understand the inhibition of viral infection by the carrageenan of *Halymenia floresii*. 

### 3.3. Carrageenan from Halymenia floresii 

The order Halymeniales have been reported to present hybrid carrageenans and complex galactans. The genus *Halymenia* is generally composed of λ-carrageenan with a high content of sulfate groups, as reported for *H. durvillei* with 44% [60], and for *H. floresii* [42] with 42.37% dw in native carrageenan extracted by hot water. In our study, the lower content of sulfate groups (~20%) compare to the study of [42], could be related to the origin of the biomass collected (seaweed stranding vs. wild banks). The FTIR and the ^1^H NMR spectrometry, coupled with the solubility test in 0.3 M KCl and the very low content of 3,6-anhydrogalactose, allowed us to infer that the main sulfated polysaccharide in *H. floresii* was λ-carrageenan since commercial -κ and ι-carrageenan were insoluble in KCl. Furthermore, gelation of -κ and ι-carrageenan was induced by the presence of KCl; on the contrary, native λ-carrageenan from *H. durvillei* did not produce gel in the presence of potassium solutions [60]. The content of non-galactose components, such as fucose, arabinose, galactose, and xylose, were also detected in the native λ-carrageenan from *Halymenia durvilei*; the authors reported that only D-xylopyranoses and d-galactopyranoses branched units were assigned to the polysaccharide structure [60]. The high viscosity of the samples did not allow ^13^C NMR analyses in *H. floresii*, and the non-identified chemical shifts on the ^1^H NMR suggested the occurrence of other types of carrageenan. 

The calculated 732.9 kDa for the used commercial carrageenan are in accordance with reported commercial available food-grade carrageenan with Mw distribution ranging from 400 to 990 kDa [61]. The semi-refined carrageenans of *H. floresii* of both EAE and HWE showed higher Mw (1200–1500 kDa) than the commercial carrageenan, even after the ion exchange purification process. Lower Mw was calculated for Fraction 2 (909 and 1004 kDa), because the ion exchange purification may exclude molecules with high molecular weight in F2. In our results, the semi-refined sulfated polysaccharide extracted by the enzyme presented a higher anti-herpetic activity which could be related to the small peak around 4.4 kDa observed in the SEC chromatogram, and as mentioned before, some LMWC can enhance the antiviral activity. Depolymerization studies are presently underway to evaluate the antiviral activity of this active anti-herpetic carrageenan on HSV infection, including both HSV-1 and -2 acyclovir-resistant strains.

### 3.4. Seaweed Stranding as Renewable Source for Potential Antivirals Polysaccharides 

Seaweed blooms and stranding events are still increasing worldwide as the inherent economic and social problems, remarking the urgency of developed and applied strategies to manage it. In light of the results presented, our research suggested a potential sector to be explored further, since seaweeds were exclusively collected from blooms and stranding events. The production of antiviral sulfated polysaccharides against HSV, through an environmentally sustainable process (EAE), demonstrated the importance of this under-utilizable bio-material and could enhance the add-value of seaweed stranding and blooms. Although high molecular weight is a barrier for SPs to get into the bloodstream, it is possible that low molecular weight oligosaccharides could penetrate into the blood or penetrate the skin. Thus, the potential of these SPs could be in the production of inexpensive oligosaccharides which can be used as topical creams for therapeutic use, since biomass can be easily found in nature. However, the use of biomass from strandings, according to Robledo et al. [62], first, must be necessary to define technical and ecological measures to forecast an event, and so the authors propose a key strategy including exploratory, valorization, and management, which allow to identify the potential applications and/or ecological services to develop its exploitation and mitigation in a region and global scale. The authors highlight the importance of the variation in the abundance and chemical composition of stranded biomass. In this sense, *Ulva* sp., *Sargassum muticum*, and *Solieria chordalis* have been identified as highly proliferative species producing huge stranding events in the French Brittany coasts [33]. While *Halymenia floresii* stranding is common during the north wind season in Yucatan, Mexico [41], this species has previously been identified with high cultivation potential. Its cultivation, under integrated multi-trophic aquaculture (IMTA) systems, has shown the feasibility to produce biomass, and in a sustainable way, is potentially rich in carbohydrates, proteins, fatty acids, and secondary metabolites, such as mycosporine-like amino acids [63]. Even, studies on its life-cycle have been successful in producing monospores and acrochaetial stage as seed material [41]. Furthermore, it seems suitable that *H. floresii* could be integrated with a sustainable bio-refinery approach with the IMTA system in order to improve the recovery of different products following the succession of extraction steps, such as was proposed for the IMTA-cultivated *S. filiformis*, in which different valuable products were extracted by EAE and MAE, including ι-carrageenan with anti-herpetic activity [64].

In this research, we have proven the antiviral activity of SPs against HSV-1, which suggested a potential added-value product for seaweed stranding. The intervention of more scientific research to integrate a bio-refinery model to upgrade the biomass as a whole benefit and the involvement of government and companies to support and develop this seaweed research will provide new opportunities for developing nations to grow. Furthermore, as was proposed by Bourgougnon et al. [65], the sustainable development goals of the universal program “2030 Agenda for Sustainable Development” were approached by proposing solutions based on algae and on key actors who utilize algae in healthy functions of the global ecosystem, thus contributing to job creation and economic growth. 

## 4. Materials and Methods

### 4.1. Algal Material

All algal biomass was collected exclusively from algal stranding events. On the littoral zone of Saint Gildas de Rhuys in Brittany, France, the red seaweed *Solieria chordalis* (Rhodophyta, Gigartinales), the green *Ulva* sp. (Chlorophyta, Ulvales), and the brown *Sargassum muticum* (Ochrophyta, Fucales) were collected. While, on the coast of Sisal in Yucatan, Mexico, *Halymenia floresii* (Rhodophyta, Halymeniales) were collected. The freshly collected biomass was cleaned, freeze-dried, and milled until powder (raw dry biomass).

### 4.2. Extraction of Sulfated Polysaccharides

Dry powder was depigmented and defatted twice in a Soxhlet extractor by 96% ethanol (EtOH), methanol:chloroform (MeOH:CHCl_3_, 1:1), and 100% acetone, 3 h per solvent. The powder was dry under the hood at room temperate (RT) overnight for polysaccharide extractions. 

**Figure 7:**
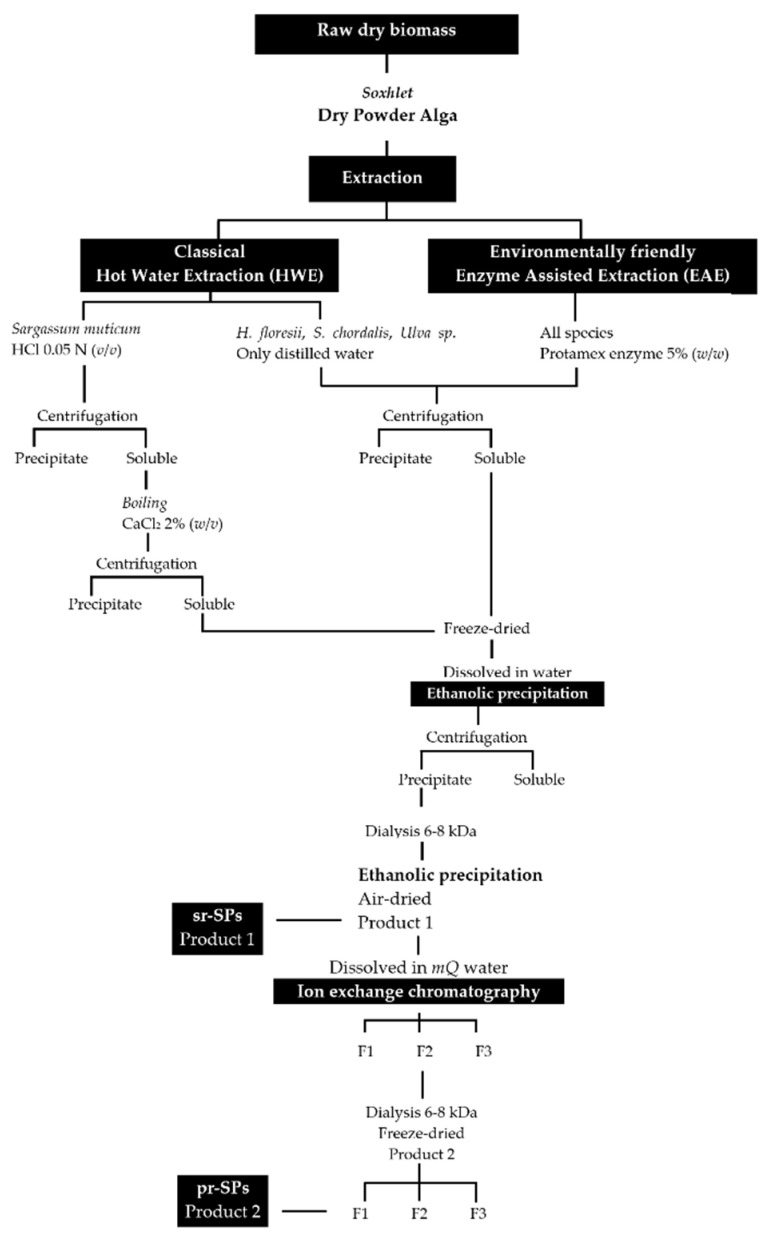
Extraction and purification procedures for sulfated polysaccharides from *Halymenia floresii*, *Solieria chordalis*, *Ulva* sp. and *Sargassum muticum*. sr-SPs: semi-refined sulfated polysaccharide; pr-SPs: purified sulfated polysaccharide.

Extractions were done following an environmentally friendly enzyme-assisted extraction (EAE) and were compared with the classical hot water extraction (HWE). The enzyme endo-protease Protamex^®^ (Novozymes, Bagsværd, Denmark) was initially added at 5.0% (*w*/*w*) to perform the EAE. The extraction for *S. muticum* with hot water (HWE) was prepared with HCl at a final concentration of 0.05 N in order to solubilize the alginic acids. All extractions were performed in triplicate using modular laboratory reactors (Mac Technology, Fontenay-Trésigny, France) on 20 g dry weight (dw) of sample and 500 mL of distilled water, during 3 h at 50 °C and stirring at 350 rpm. Afterward, the temperature was increased to 90 °C for 15 min to denature the enzyme. Extractions were filtrated through cheesecloth and the solubilized material was centrifuged (5000× *g* 10 min) to remove insoluble residues. The insoluble residues were washed twice with 100 mL of distilled water and centrifuged again (5000× *g* 10 min); all soluble parts recovered were combined. The soluble part of *S. muticum*, both EAE and HWE were treated with solid calcium chloride (CaCl_2_) at the final concentration of 2% to promote alginate precipitation, boiled 2 h at 70 °C and shaking, and after cooled down the sticky precipitate was discarded and the supernatant was centrifuged again (5000× *g* 10 min). All soluble fractions were frozen, lyophilized, weighed, and stored at −20 °C for further use. The lyophilized material was dissolved in distilled water and polysaccharides were precipitated with four volumes of 99% cold EtOH (*v*/*v*) at 4 °C overnight. Precipitates were recovered by centrifugation (7000× *g* 20 min), washed out with absolute EtOH and acetone, air-dried under the hood at RT, and milled until a fine powder. The powder was re-suspended in distilled water at a final concentration of 20 mg/mL and extensively dialyzed against distilled water using 6–8 kDa molecular weight-cutoff (MWCO) membrane (Spectra/Por, Fisher Scientific, Illkirch, France) for 3 days at 4 °C. Polysaccharides were recovered again with EtOH as described above until they became dry powder (Figure 7). These samples were denoted semi-refined sulfated polysaccharides (sr-SPs).

### 4.3. Purification of Semi-Refined Sulfated Polysaccharides (sr-SPs)

**Figure 8:**
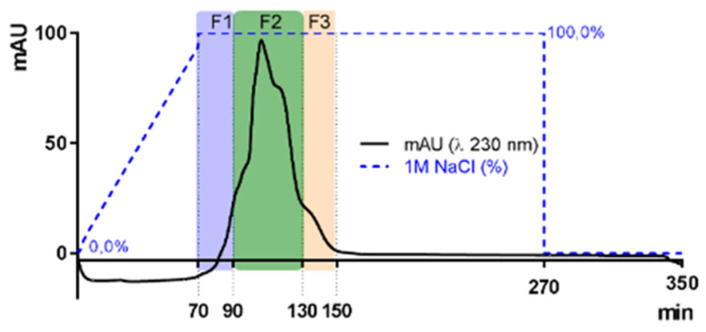
Elution curves of sulfated polysaccharides from *Halymenia floresii* trough ion exchange resin DEAE-Sepharose fast-flow for purification. Fractions were obtained according to elution time F1, F2, and F3. The blue dotted line represents the percentage of 1M NaCl as eluent.

The sr-SPs were purified by ion exchange chromatography. Where 300.0 mg of sr-SPs were dissolved in MQ water, filtered at 1 µm (Chromafil Xtra, Macherey-Nagel, Duren, Germany), and loaded to an XK 26 Pharmacia column (500 × 600 mm) manually packed with DEAE-Sepharose fast-flow anionic resin (GE Healthcare, Uppsala, Sweden). The column was coupled in an HPLC system with a diode array detector (Dionex U3000 Thermo, Germering, Germany). The column was initially eluted with distilled water at a flow rate of 4.0 mL/min, followed by an increasing concentration of NaCl solution until 100% 1M NaCl was reached at minute 70 min. Afterward, an isocratic elution of 1M NaCl was maintained until 270 min and returned to 100% distilled water until 350 min. Peaks were recorded by Chromeleon 6.8 software (Thermo Scientific, Illkirch, France) at 230 nm (e.g., Figure 8). Three fractions F1, F2, and F3 (80, 160, and 80 mL) were obtained, dialyzed, and lyophilized. Samples were denoted as purified sulfated polysaccharides (p-SPs). All purifications were done in triplicate.

### 4.4. Screening for Antiviral Activity In Vitro and Cytotoxicity of Algal Polysaccharides 

Vero cells (African green monkey kidney cell line, ATCC CCL81) were used for culturing HSV-1. The cells were grown in MEM supplemented with 8% fetal calf serum (FCS, Eurobio) and 1% of antibiotics PCS (10,000 IU penicillin mL-1, 25,000 IU colimycin mL^−1^, 10 mg streptomycin mL^−1^, Sigma). All the cells were cultured at 37 °C in a humidified atmosphere supplied with 5% CO_2_. HSV-1 (wild-type strain 17, sensitive to acyclovir) was obtained from Pr. David Boutolleau (Virology Department, Hospital Pitié-Salpétrière, France). Microplates of 96-well flat-bottom TC Falcon (Corning, France) were prepared by adding 100 μL per well of cellular suspension (3.5 × 10^5^ cells/mL); 50 μL per well of acyclovir ([9-(2-hydroxyethoxymethyl)guanine] as a reference inhibitor (Thermo, France) with five concentrations (5.0, 1.0, 0.5, 0.1 and 0.05 μg/mL). Fifty μL of SPs, per well, with five concentrations ranging from 1.0 to 200 μg/mL were added. Cells were infected with 50 μL/mL per well of viral suspension, at a multiplicity of infection (MOI) of 0.01 ID_50_/cells. Simultaneously, cytotoxicity was tested by adding 50 μL of MEM to cells’ suspension with acyclovir or polysaccharide. A virus control and cells control were also tested. Microplates were incubated for 72 h at 37 °C and 5% CO_2_. After incubation, all microplates were examined under a phase-contrast microscope to confirm the presence of the cell monolayers and discard a possible detachment of cells to the wells; and to observe possible alterations on cell morphology (swelling, shrinkage, or granularity). Then, 50 μL per well of neutral red was added, and the microplates were incubated for 45 min. The dye was eliminated, and the wells were washed out with 200 μL PBS buffer (two times). Finally, 100 μL of ethanol citrate was added to burst the living cells, and thus the neutral red was released and read by spectrophotometry. Both antiviral activity and cytotoxicity were evaluated by the neutral red dye method from their optical density (OD) at 540 nm [66]. The OD is related directly to the percentage of viable cells, which is inversely related to the cytopathic effect (CPE). The 50% effective antiviral concentration (EC_50_ μg/mL) was the concentration that achieved 50% protection of virus-infected cells from virus-induced destruction. A linear regression was determined based on cell control (0% CPE) and virus control (100% CPE), data were expressed as a percentage of protection (%P), and was calculated as:

%P = [((OD_t_)_virus_ − (OD_c_)_virus_)/((OD_c_)_MOCK_ − (OD_c_)_virus_)] × 100, (1)

where (OD_t_)_virus_, (OD_c_)_virus_, and (OD_c_)_MOCK_ were the OD of the tested sample, virus control, and the mock-infected control, respectively. While cytotoxicity by cell viability, was defined as the concentration that reduced the OD of treated cells to 50% from untreated cells (CC_50_) and was expressed as the percentage of destruction (%D) and calculated as follow:

%D = [(OD_c_)_C_ − (OD_c_)_MOCK_/(OD_c_)_C_] × 100, (2)

where (OD_c_)_C_ are the control cells and (OD_c_)_MOCK_ are the OD values of the mock-infected control [67]. Each OD value was the average of four technical repetitions (four wells) per microplate, except virus and cell control which was 8 wells. The assay was done in duplicates (*n* = 2).

A linear regression was used to observe the antiviral activity (EC_50_ μm/mL) as a function of the sulfate content (*r*^2^ coefficient) for the different fractions of *H. floresii SPs*, both EAE and HWE.

### 4.5. Antiviral Activity at Different Treatment Schemes from Selected Polysaccharides

The antiviral activity from *H. floresii*, both EAE and HWE semi-refined polysaccharides, and the purified Fractions 2 (F2) from EAE of *S. chordalis* and *S. muticum*, were evaluated at different treatment schemes [68] since these samples showed the higher antiviral activity. All assays were done in 96-well microplates, each treatment scheme was performed in duplicate (*n* = 2) using the five concentrations of the SPs above-mentioned, and virus and cell controls were added simultaneously. The microplates were evaluated and EC_50_ values were calculated as earlier detailed [66].

Pre-treatment of the cells with SPs. Microplates were prepared by adding 100 μL of cellular suspension (3.5 × 10^5^ Vero cells/mL), 50 μL of acyclovir (5, 1, 0.5, 0.1 and 0.05 μg/mL) and 50 μL of SPs (200, 50, 10, 5 and 1.0 μg/mL), and the wells were completed to 200 μL (total volume) with MEM. After 24 h of incubation at 37 °C, 5% CO_2_, microplates were washed with 100 μL of PBS. Fifty μL of viral suspension were added, the wells were completed with MEM in a total volume of 200 μL and incubated for 72 h (37 °C, 5% CO_2_).

Pre-treatment of the virus with SPs. Microplates were prepared with 100 μL of cellular suspension and incubated 24h (37 °C, 5% CO_2_). Before the cell infection, viral suspensions were incubated with an equal volume of the corresponding concentrations of SPs or acyclovir for one hour (37 °C, 5% CO_2_). Afterward, 100 μL of each mixed suspension were added and incubated for 72 h.

The viral adsorption assays. The cellular suspension was incubated for 24 h at 37 °C, 5% CO_2_. Cells were infected following three different treatments: TA, TB, and TC. During TA, cells were infected in the presence of the SPs or acyclovir, then incubated for 1 h at 4 °C to promote the viral adsorption; afterward, the SPs, acyclovir, and the non-adsorbed virions were eliminated by rinsing the monolayer of Vero cells with 100 μL of PBS. Then, 200 μL of MEM was added, and the microplate was incubated for 72 h, at 37 °C, 5% CO_2_. In TB, the cells were exposed to the virus for 1 h at 4 °C, then the microplates were rinsed with PBS, and the SPs, acyclovir, and MEM were added and incubated (24 h, 37 °C, 5% CO_2_). Lastly in TC, the SPs, and acyclovir were added before and after the viral adsorption period (1 h at 4 °C) and incubated (72 h, 37 °C, 5% CO_2_).

The post-infection assays. The monolayers of cells were infected with the virus at 37 °C and 5% CO_2_, and the SPs and acyclovir were added at the same time (0 h), and after 1, 2, 3 and 5 h of the cell infection followed by 72 h of incubation (37 °C, 5% CO_2_).

### 4.6. Biochemical Composition of Polysaccharides

The purified fractions and the semi-refined polysaccharide from *Halymenia floresii* were analyzed for neutral sugars using the phenol sulphuric acid method [69], 3,6-anhydrogalactose [18]; sulfate groups [70], uronic acids [71], and protein content using the Bicinchoninic Acid assay [72]. The monosaccharide composition was done using a High-performance anion-exchange chromatography (HPAEC) coupled with a pulsed amperometric detection (PAD) (Thermo Dionex, Sunnyvale, CA, USA) according to [73]. Where 25 μL of the sample was injected on a CarboPac PA-1 column (4.6 × 250 mm) connected to a CarboPac pre-column (Thermo Dionex, Illkirch, France). The elution was carried out isocratically, keeping the mobile phase for 30 min with 82% milli-Q water and 18% 0.1M NaOH, followed by a gradient from minutes 31 to 35 with 100% of 0.1M NaOH + 1 M NaOAc. Finally, from minutes 36 to 80, elution was done with 82% milli-Q water and 18% 0.1M NaOH. The column temperature was fixed at 30 °C and the flow was monitored with a PAD (gold) operated at a sensibility of 1000 nA. Peaks were detected by Chromeleon 6.8 software (Thermo Scientific, Illkirch, France). All solvents were previously degassed with helium gas. Monosaccharides were identified and quantified based on their standard curves at different concentrations (3–125 ppm), mannitol, fucose, glucosamine, rhamnose, galactose, glucose, mannose, xylose, fructose, ribose, and glucuronic acid. Deoxyribose was used as an internal standard. Results were expressed as total content: µg of monosaccharides per mg of dry weight (μg/mg dw); and as individual content in percentage of each monosaccharide from the total content (% of total content). Representative chromatograms are presented in Supplementary Figure S2.

### 4.7. Characterization of H. floresii Polysaccharide

Semi-refined polysaccharides of *H. floresii* both EAE and HWE were partially characterized since they showed the higher antiviral activity. The polysaccharide molecular weight (MW) was determined by two methods: by size-exclusion chromatography (HPSEC) and from their intrinsic viscosity, in order to found reliable results because the MW was calculated by extrapolation. HPSEC analysis was done using a UHPLC (U3000 Thermo, Scientific, Illkirch, France) equipped with a differential refractometry Iota 2 (Precision Instrument, Marseille, France). Polysaccharide samples and dextran standards were prepared in 0.1 M sodium nitrate (NaNO_3_) at a final concentration of 1 mg/mL and filtered with a 0.45 μm syringe filter (Sartorius Stedim biotech, Goettingen, Germany). 100 μL of the sample were injected in a column TSKgel^®^ G6000PWXL (7.8 mm × 30.0 cm, 13 μm), preceded by a guard column TSKgel PWXL (6.0 × 40 mm, 12 μm, Tosoh Bioscience, Griesheim, Germany). Elution was performed isocratically during 15 min with 0.1 M NaNO_3_ solution (vacuum-filtered through 0.45 μm and degassed) at a rate of 1.0 mL/min. The column temperature was kept constant at 30 °C. Detection peaks were done using a refractive index detector and chromatograms were analysed by Chromeleon 6.8 software (Thermo Scientific, Illkirch, France). The MW of polysaccharides were estimated according to Gómez-Ordónez et al. [61], based on the regression equation (y = −0.392x + 6.512; *r*^2^ 0.9930) of the standard calibration curve by plotting the logarithm of the molecular weight versus retention time. As standards used were dextran sulfate: 1, 5, 12, 25, 50, 80, 150, 270, 410, 670 kDa, and the blue dextran 2000 kDa (Sigma-Aldrich, Missouri, MO, USA).

The estimation of the weight average molecular mass (AMw) by intrinsic viscosity was done according to [74]. Polysaccharides were prepared in triplicates in 0,01 M NaCl at the final concentrations of 0.25, 0.12, 0.062, 0.031, 0.0152 and 0.00% (*w*/*v*), heated at 60 °C, 20 min and constant stirring. The dynamic viscosities (η) versus the shear rates (γ) were obtained from a controlled rate viscometer RotoVisco 1 (Thermo Haake, Karlsruhe, Germany) with a 35 mm plate diameter and 2° cone angle. Furthermore, 500 μL of the sample was applied to the plate and closed at 105 μm distance between plates, the shear rate ranging from 1–1000 s^−1^, and the temperature was kept at 30 °C. Data were collected and processed using the integrated software Haake RheoWin v3. The AMw was calculated according to the Mark–Houwink equation:

[η] = K × MW^α^, (3)

where α and K are the coefficients obtained from [74], and the intrinsic viscosity [η] was calculated from the linear equation obtained by plotting the polysaccharide concentration versus the reduced viscosity. The reduced viscosity (η_red_) was calculated as:

η_red_ = (η − η_0_)/(η_0_ × *c*), (4)

where η_0_ and η are the dynamic viscosities of the dissolvent and the polysaccharide, respectively; and *c* is the polysaccharide concentration. According to the extrapolation to zero, the intrinsic viscosities [η] were used in Equation (3). 

The determination of basic structural polysaccharide composition was done through the Fourier Transformed Infrared (FTIR) spectroscopic analysis, using the Nicolet iS5 spectrometer (Thermo, Madison, WI, USA), equipped with a universal attenuated total reflectance (ATR) sampling device containing a diamond crystal. Approximately 100 mg of dry sample, both EAE and HWE semi-refined polysaccharide, was placed directly into the sampling device and pressed towards the diamond. Samples were recorded in transmission mode at room temperature from 500 to 4000 cm^−1^, with 16 scans and a resolution of 4 cm^−1^. Background spectra of air were scanned before to analyze samples. The FTIR spectra were acquired and processed by Omnic 9.3.32 software (Thermo Scientific, Illkirch, France). The spectra values are presented as the average of three counts. The commercial carrageenans (SKW Biosystems, Boulogne, France): lambda (Satiagum BDC 20), kappa (Stiagel ME 5), and iota (Siatagel DF 52) were used for FTIR comparison with the polysaccharides from *H. floresii*. The FTIR spectra for the commercial kappa and iota-carrageenans (data not shown) were different from the commercial lambda-carrageenan and the polysaccharides from *H. floressi*, which were very similar to each other. Second-derivatives of FTIR spectra for the polysaccharide from *H. floresii* were included to improve the resolution, and derivation was done through the Savitzky–Golay algorithm with 25 smoothing points and a third-degree polynomial equation [61].

Nuclear magnetic resonance (^1^H NMR) spectra were recorded at 343 or 353 °K on a Bruker Avance III HD 500 spectrometer (Bruker, Wissembourg, France) equipped with an indirect 5 mm probe head BBO 1H/{BB}. Samples were solubilized in 700 μL of D2O 99.96%. The spectra were performed according to Bruker’s pulse programs with standard pulse sequence, delay of 2 s, and a 30° pulse. Chemical shifts were expressed in ppm relative to tetramethylsilane (TMS) as an external reference.

The solubility of the carrageenans in 0.3 M potassium chloride (KCl) solution was evaluated according to [42]. The commercial carrageenans lambda (Satiagum BDC 20), kappa (Stiagel ME 5), and iota (Siatagel DF 52), and the semi-refined and purified sulfated polysaccharides (0.1% powder), both EAE and HWE from *Halymenia floresii*, were mixed with 0.3 M KCl solution. The mixtures were continuously stirring for 1 h at room temperature, after 2 h, left to stand, and mixtures were filtered and observations were done to see whether carrageenan had solubilized. Four volumes of isopropanol were added and after 1 h the mixtures were centrifuged (10 min, 5000 rpm) in order to re-precipitate the carrageenans.

### 4.8. Statistical Analysis

All results were presented as mean of n replicated ± standard deviation. According to the respective experiment (specified in each table or figure), the significant differences were analyzed by the T-Student test (two tails) or by one-way ANOVA with Tukey HSD. However, if data failed for normality and homoscedasticity of variance tests, a non-parametric Kruskal–Wallis H test with multiple comparisons was applied. All analyses were performed using Infostat 2020 Student version, with a significance level α established at 0.05 for all tests. Graphics were elaborated using GraphPAD Prism and SigmaPlot.

## 5. Conclusions

Overall, the antiviral activities from polysaccharides, which included ulvans, fucans, and galactans, were determined by the differences in the structural features, sulfation degree, and distribution of sulfate groups in the molecule, the molecular weight, and the constitution of monosaccharides. A potent antiviral polysaccharide was obtained from *Halymenia floresii* after EAE, an eco-friendly procedure that allowed increasing the yields of extraction compared to the hot water extraction. Further studies are underway to identify the possible hybrids presented in the carrageenan and to evaluate the antiviral activity of LMWC produced from *H. floresii*. We demonstrated, at least in part, that biomass from blooms and stranding events is adequate to produce sulfated polysaccharides with antiviral activity.

## Figures and Tables

**Table 1:** Antiviral activity of the sulfated polysaccharides against HSV-1 at different treatment schemes: the virus adsorption assay (TA, TB, TC) and the post-infection assay. Antiviral activity expressed as EC_50_ (µg/mL) and at viral suspension at MOI 0.001 ID_50_/cells.

Assay	*H. floresii* EAE	*H. floresii* HWE	*S. chordalis* *F2*	*S. muticum* *F2*	Acyclovir (Control)
Virus adsorption					
TA	0.38 ± 0.06	0.60 ± 0.01	56.7 ± 2.3 *	83.6 ± 3.3 *	0.42 ± 0.04
TB	1.76 ± 0.2	7.65 ± 0.4	181 ± 9.1	143 ± 4.5	0.38 ± 0.04 *
TC	0.18 ± 0.02	0.74 ± 0.03 *	31.5 ± 3.2 *	54.8 ± 4.6 *	0.09 ± 0.03
Post-infection					
0 h	1.28 ± 0.4	4.14 ± 0.4	46.5 ± 4.8	36.5 ± 0.9	0.31 ± 0.08 *
1 h	2.47 ± 0.1	4.62 ± 0.03	83.7 ± 2.2	57.0 ± 2.3	0.24 ± 0.02 *
2 h	6.59 ± 0.4	5.02 ± 0.1	>200	75.5 ± 2.6	0.73 ± 0.01 *
3 h	5.42 ± 0.05	5.25 ± 0.5	>200	102 ± 5.4	1.70 ± 0.3 *
5 h	19.1 ± 0.6	22.8 ± 2.07	>200	>200	0.83 ± 0.1 *

EAE: Enzyme-assisted extraction; HWE: Hot water extraction. Data are the mean ± standard deviation (*n* = 2). * Significance difference (*p* < 0.05) by row, acyclovir vs. polysaccharide sample (*t*-test for independent samples, two tails).

**Table 2:** Biochemical composition (% of dry weight) of purified sulfated polysaccharides (p-SPs) Fraction 2 obtained by enzyme-assisted extraction (EAE) or hot water extraction (HWE).

Species	Neutral Sugars		Sulfate Groups		Uronic Acids		Protein		3,6-AG	
	EAE	HWE	EAE	HWE	EAE	HWE	EAE	HWE	EAE	HWE
*H. floresii*	36.9 ± 0.04 ^a^	37.0 ± 0.05 ^a^	9.4 ± 0.1^a^	10.1 ± 0.02 ^b^	3.16 ± 0.05 ^a^	3.13 ± 0.04 ^a^	1.18 ± 0.03 ^a^	1.12 ± 0.03 ^a^	0.6 ± 0.04 ^a^	0.5 ± 0.02 ^a^
*S. chordalis*	22.8 ± 0.7 ^a^	23.7 ± 0.5 ^a^	15.4 ± 0.2^a^	13.5 ± 0.4 ^b^	7.6 ± 0.1^a^	6.7 ± 0.09 ^b^	7.5 ± 0.1 ^a^	6.2 ± 0.02 ^b^	8.4 ± 0.3 ^a^	8.8 ± 0.08 ^a^
*Ulva* sp.	23.1 ± 0.8 ^a^	28.4 ± 0.5 ^b^	5.9 ± 0.1^a^	5.7 ± 0.1 ^a^	17.5 ± 0.4 ^a^	17.1 ± 0.02 ^a^	5.0 ± 0.03 ^a^	3.5 ± 0.3 ^b^	ND	ND
*S. muticum*	25.0 ± 0.8 ^a^	27.0 ± 0.8 ^a^	12.9 ± 0.08 ^a^	11 ± 0.4 ^b^	15.6 ± 0.7 ^a^	16.1 ± 0.5 ^a^	6.0 ± 0.05 ^a^	3.1 ± 0.07 ^b^	ND	ND

3,6-AG: Anhydrogalactose; Data are means ± SD (*n* = 3). ND: not determined. Different letters are significantly different (*p* < 0.05) between EAE and HWE by biochemical group (one-way ANOVA, Tukey HSD test *p* < 0.05).

**Table 3:** Biochemical composition of semi-refined sulfated polysaccharides (sr-SPs) from *Halymenia floresii* obtained by enzyme-assisted extraction (EAE) or hot water extraction (HWE).

Sample	Carbohydrates	Sulfate Groups	Uronic Acids	Protein	3,6-AG
EAE	24.7 ± 1.6	17.2 ± 1.9	1.9 ± 0.25	2.7 ± 0.3	1.0 ± 0.02
HWE	24.9 ± 2.1	18.6 ± 2.3	2.1 ± 0.3	2.9 ± 0.2	1.2 ± 0.01

3,6-AG: 3,6-Anhydrogalactose. Data are the mean ± standard deviation (*n* = 3).

**Table 4:** Weight average molecular mass (AMw) and molecular weight (Mw) calculated for the λ-carrageenan semi-refined from *H. floresii* obtained by enzyme-assisted extraction (EAE) or hot water extraction (HWE), and for the λ-commercial sample from SATAgum, France.

Sample	AMw (kDa) ^1^	MW (kDa) ^2^
EAE	1202.8 ± 60.1	4.4 ± 0.6 ^3^
HWE	1569.8 ± 40.01	ND

^1^ Weight average molecular mass (AMw) calculated by the intrinsic viscosity. ^2^ Molecular weight (Mw) determined by size-exclusion chromatography (HPSEC). ^3^ Peak comprised into the ranged of the standards. ND: not detected. Data are the mean ± standard deviation (*n* = 3).

## Data Availability

The data presented in this study are available on request from the corresponding author.

## Supplementary Materials

The following supporting information can be downloaded at: https://www.mdpi.com/article/10.3390/md20020116/s1, Figure S1. Yields of sulfated polysaccharides obtained by enzyme-assisted extraction (EAE), or hot water extraction (HWE), Table S1. In vitro antiviral activity (EC_50_ μg/mL) of sulfated polysaccharides against HSV-1, Table S2. Cytotoxicity (percentage of destruction on Vero Cells) of the sulfated polysaccharide, sr-SPs, p-SPs fractions F2 and FCSP, Table S3. Biochemical composition (% of dry weight) of purified sulfated polysaccharides (p-SPs), Fractions F1 and F3, Table S4. Profile of monosaccharides of the purified sulfated polysaccharides (p-SPs) Fraction F2, Figure S2. Chromatograms for identification of the monosaccharide composition by HPAEC.
