# Supplementary material for: Sulfated Polysaccharides from Seaweed Strandings as Renewable Source for Potential Antivirals against Herpes simplex Virus 1
Source: Mar Drugs. 2022 Feb 1;20(2):116. doi: 10.3390/md20020116 (PMC8878361; doi:10.3390/md20020116)
Supplement: Supplementary file 1 [file marinedrugs-20-00116-s001.zip › marinedrugs-1477613-supplementary.pdf]

Article

Sulfated Polysaccharides from Seaweed Strandings as Renewable Source for Potential Antivirals against *Herpes simplex* Virus 1

Hugo Pliego-Cortés [1], Kévin Hardouin [1], Gilles Bedoux [1], Christel Marty [1], Stéphane Cérantola [2], Yolanda Freile-Pelegrín [3], Daniel Robledo [3], and Nathalie Bourgougnon [1,*]

Supplementary Material

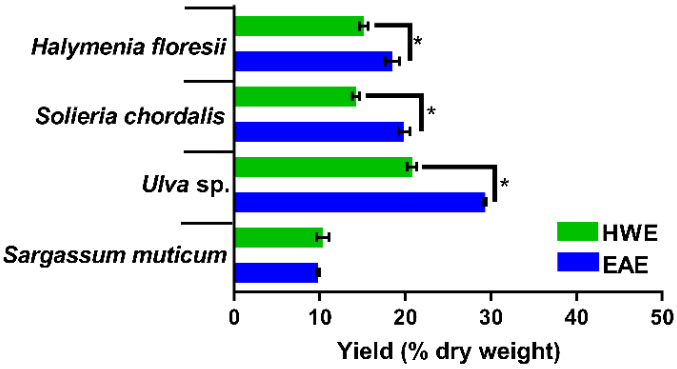

Figure S1. Yields of the semi-refined Sulfated Polysaccharide (sr-SPs) obtained by Enzyme-Assisted Extraction (EAE), or Hot-Water Extraction (HWE). The yields were expressed as percentage of seaweed dry weight (% dw), after EtOH and dialysis isolation. Data are means ± SD (n=3). *Denote significant differences (one-way ANOVA, Tukey HSD test p<0.05).

Table S1. *In vitro* antiviral activity (EC_{50} µg/mL) of Sulfated Polysaccharides against HVS-1 after 72 h infection at MOI 0.001 ID50/cells, of the semi-refined Sulfated Polysaccharide (sr-SPs), and purified Sulfated Polysaccharide (p-SPs) fractions F1, F2 and F3

**Specie**		***sr-SPs***	***F1***	***F2***	***F3***
Halymenia floresii	EAE	0.68 ± 0.03*	27.5 ± 2.05	3.3 ± 0.3	3.84 ± 0.08
	HWE	1.24 ± 0.06*	30.3± 3.1	3.94 ± 1.03	7.68 ± 0.4
Solieria chordalis	EAE	60.7 ± 7.7	39 ± 0.41	18.4 ± 1.1*	>200
	HWE	73.5 ± 2.4	39.1 ± 1.5	19.3 ± 1.05*	>200
Ulva sp.	EAE	>200	>200	169.5 ± 3.6	>200
	HWE	>200	>200	179.4 ±10.1	>200
Sargassum muticum	EAE	68.8 ± 3.4	40.5 ± 1.4	27.2 ± 0.47*	80.3 ± 1.5
	HWE	73.2 ± 4.22	37.4± 3.0	20.05 ± 0.55*	76.5 ± 1.0
Acyclovir		0.45 ± 0.01	0.44 ± 3.0	0.59 ± 0.04	0.5 ± 0.02

Data are the mean ± Standard deviation (n=2). *Significance difference ($p < 0.05$) by row from EAE and HWE and by column from FCSP (One-way non-parametric ANOVA Kruskal-Wallis, pairwise and multiple comparisons, respectively).

Table S2. Cytotoxicity (percentage of destruction on Vero Cells) of the semi-refined Sulfated Polysaccharide (sr-SPs), and purified Sulfated Polysaccharide (p-SPs) of fractions F2.

**Species**	**Fraction**	**Cytotoxicity Polysaccharide concentration (µg/mL)**						
		**1000**	**500**	**200**	**50**	**10**	**5**	**1**
Halymenia floresii EAE	sr-SPs	7.5 ± 0.5	7.3 ± 0.3	5.3 ± 0.1	5.3 ± 0.01	5.7 ± 0.1	4.3 ± 0.2	3.5 ± 0.3
	F2	6.3 ± 0.5	7.0 ± 0.2	6.1 ± 0.2	6.0 ± 0.3	4.1 ± 0.3	5.1 ± 0.1	3.9 ± 023
Halymenia floresii HWE	sr-SPs	5.5 ± 0.6	6.0 ± 0.2	1.8 ± 0.01	2.2 ± 0.1	2.6 ± 0.1	3.8 ± 0.2	3.6 ± 0.1
	F2	6.1 ± 0.4	5.8 ± 0.5	3.2 ± 0.2	3.0 ± 0.3	1.9 ± 0.2	2.5 ± 0.2	2.1 ± 0.1
Solieria chordalis EAE	sr-SPs	9.5 ± 0.4	7.8 ± 0.2	2.5 ± 0.1	2.9 ± 0.1	1.9 ± 0.1	2.0 ± 0.1	1.1 ± 0.07
	F2	8.8 ± 0.2	8.1 ± 0.4	3.2 ± 0.01	3.7 ± 0.1	2.6 ± 0.2	3.1 ± 0.1	2.3 ± 0.1
Solieria chordalis HWE	sr-SPs	7.5 ± 0.5	7.0 ± 0.2	2.2 ± 0.2	2.5 ± 0.2	1.9 ± 0.3	2.2 ± 0.2	1.1 ± 0.05
	F2	8.1 ± 0.2	6.8 ± 0.3	3.0 ± 0.01	3.7 ± 0.2	2.3 ± 0.1	3.2 ± 0.3	2.5 ± 0.2
Ulva sp. EAE	sr-SPs	/	/	6.0 ± 0.1	5.7 ± 0.2	2.9 ± 0.1	3.2 ± 0.2	1.6 ± 0.01
	F2	/	/	5.8 ± 0.3	6.1 ± 0.1	3.6 ± 0.3	3.0 ± 0.1	1.3 ± 0.03
Sargassum muticum EAE	sr-SPs	/	/	3.9 ± 0.3	3.7 ± 0.1	2.3 ± 0.2	2.2 ± 0.1	1.6 ± 0.05
	F2	/	/	5.1 ± 0.2	3.9 ± 0.3	2.7 ± 0.1	1.7 ± 0.01	1.1 ± 0.01
Acyclovir		9.1 ± 0.5	7.8 ± 0.3	5.9 ± 0.1	7.5 ± 0.2	3.8 ± 0.1	3.6 ± 0.1	4.1 ± 0.1

Data are the mean ± Standard deviation (n=2). / tests were not performed.

Table S3. Biochemical composition (% of dry weight) of purified Sulfated Polysaccharides (p-SPs) Fractions F1 and F3 obtained by Enzyme-Assisted Extraction (EAE) or Hot-Water Extraction (HWE).

Species		Neutral sugars		Sulfate groups		Protein		3,6-AG	
		EAE	HWE	EAE	HWE	EAE	HWE	EAE	HWE
H. floresii	F1	40.9 ± 1.5[a]	39.5 ± 2.3[a]	2.1 ± 0.1[a]	1.97 ± 0.02[a]	1.65 ± 0.01[a]	1.7 ± 0.03[a]	nd	nd
	F3	45.9 ± 1.8[a]	44.5 ± 2.0[a]	2.7 ± 0.3[a]	2.5 ± 0.3[a]	1.05 ± 0.03[a]	1.1 ± 0.01[a]	nd	nd
S. chordalis	F1	34.3 ± 1.1[a]	36.2 ± 2.5[a]	2.7 ± 0.1[a]	2.5 ± 0.04[b]	8.1 ± 0.1[a]	7.2 ± 0.02[b]	9.1 ± 0.3[a]	8.9 ± 0.1[a]
	F3	40.6 ± 1.5[a]	39.8 ± 2.3[a]	<1	1.3 ± 0.05[a]	6.3 ± 0.7[a]	5.4 ± 0.8[b]	6.3 ± 0.8[a]	5.1 ± 0.3[b]
Ulva sp.	F1	45.3 ± 1.8[a]	46.1 ± 1.5[a]	2.9 ± 0.2[a]	2.8 ± 0.3[a]	3.6 ± 0.3[a]	3.9 ± 0.1[a]		
	F3	44.9 ± 2.1[a]	44.5 ± 2.3[a]	3.1 ± 0.1[a]	3.2 ± 0.1[a]	2.7 ± 0.8[a]	2.9 ± 0.3[a]		
S. muticum	F1	36.6 ± 1.1[a]	37.0 ± 0.8[a]	2.4 ± 0.08[a]	1.91 ± 0.4[b]	3.3 ± 0.02[a]	4.1 ± 0.8[b]		
	F3	42.9 ± 2.1[a]	41.7 ± 1.9[a]	1.8 ± 0.5[a]	2.1 ± 0.2[a]	3.0 ± 0.5[a]	2.8 ± 0.3[a]		

3,6-AG: Anhydrogalactose; Data are means ± SD (n=3). nd: not detected. Empty space: not determined. Different letters are significantly different ($p<0.05$) between EAE and HWE by biochemical group (one-way ANOVA, Tukey HSD test $p<0.05$).

Table S4. Profile of monosaccharides of the purified Sulfated Polysaccharides (p-SPs) Fraction F2 by DEAE-Sepharose anion exchange, obtained by Enzyme-Assisted Extraction (EAE) or Hot-Water Extraction (HWE). Individual monosaccharide as percentage (%) of total content (µg/mg dw) identified by anion exchange chromatography (HPAEC)

**Monosaccharide (% of total)**	***Ulva* sp.**		***Solieria chordalis***		***Sargassum muticum***		***Halymenia floresii***	
	**EAE**	**HWE**	**EAE**	**HWE**	**EAE**	**HWE**	**EAE**	**HWE**
Mannitol	/	/	/	/	0.47 ± 0.06	0.41 ± 0.07	/	/
Fucose	/	/	/	/	36.97 ± 1.21	33.96 ± 1.4	1.76 ± 0.21	2.16 ± 0.1
Rhamnose	72.3 ± 0.17	72.9 ± 1.16	1.82 ± 0.1	2.45 ± 0.1	0.76 ± 0.06	0.35 ± 0.1	6.27 ± 0.4	6.46 ± 0.4
Arabinose	/	/	/	/	/	/	3.16 ± 0.7	4.11 ± 0.6
Glucosamine	0.18 ± 0.01	/	3.41± 0.11	3.93 ± 0.11	2.7 ± 0.1	/	0.13 ± 0.01	0.45 ± 0.2
Galactose	6.23 ± 0.1	4.97 ± 0.1	46.59 ± 1.0	47.46 ± 0.7	26.69 ± 0.6	24.65 ± 1.6	57.8 ± 1.8	55.50 ± 1.9
Glucose	1.05 ± 0.1*	2.22 ± 0.09	16.9 ± 0.14	17.96 ± 0.1	1.6 ± 0.07	2.56 ± 0.4	0.58 ± 0.1	0.87 ± 0.2
Mannose	0.06 ± 0.001	0.33 ± 0.001	/	/	21.33 ± 1.1	28.63 ± 1.4	3.01 ± 0.12	3.16 ± 0.5
Xylose	1.02 ± 0.04	1.17 ± 0.04	0.12 ± 0.07	0.10 ± 0.02	1.54 ± 0.06	1.71 ± 0.11	0.56 ± 0.14	0.80 ± 0.04
Glucuronic acid	7.47 ± 0.09	7.8 ± 0.1	3.83 ± 0.05	4.3 ± 0.4	2.4 ± 0.06*	1.3 ± 0.06	6.5 ± 0.1	6.5 ± 0.13
Non-identified	11.5 ± 0.1	10.4 ± 0.17	27.2 ± 1.16	23.7 ± 1.2	6.41 ± 0.1	5.22 ± 0.1	20.06 ± 0.4	19.82 ± 0.1
**Total (µg/mg dw)**	**108.4 ± 10.5**	**112.5 ± 11.3**	**142.4 ± 10.18**	**147.5 ± 10.44**	**107.8 ± 10.25**	**82.92 ± 9.3**	**108.97 ± 4.12**	**114.1 ± 9.3**

/ represents not detected. Non-identified represents the sum of non-identified monosaccharides; Data are the mean of duplicates (n=2). *Significance difference ($p < 0.05$) by row between EAE and HWE for each species of seaweed (one-way non-parametric ANOVA Kruskal-Wallis, pairwise-comparison).

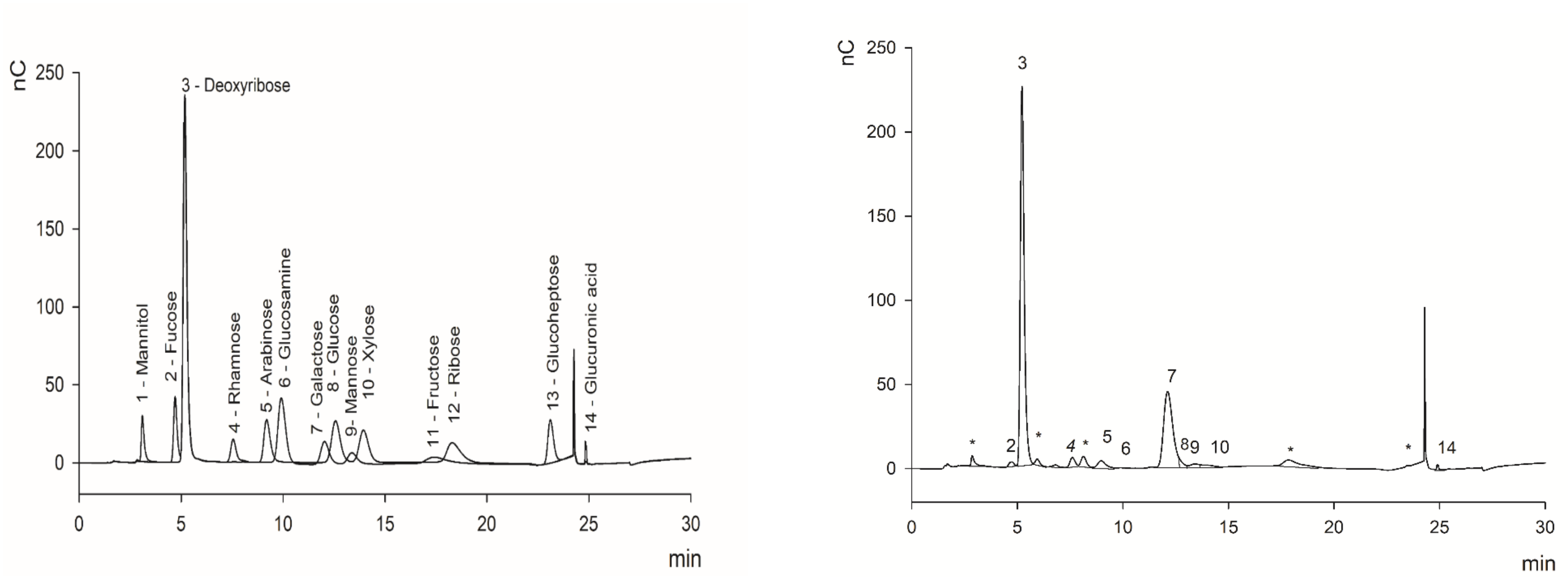

(**a**) (**b**)

Figure S2. Chromatograms for identification of the monosaccharide composition by HPAEC, (a) standard monosaccharides, (b) purified Sulfated Polysaccharide (p-SPs) Fraction 2 from *Halymenia floresii* obtained by Enzyme-Assisted Extraction. The number on the peaks corresponds to the number of the standard monosaccharide, the * denotes non-identified monosaccharide. Deoxyribose (peak 3) was used as internal standar.
